# Spectrum of Ectopic Pregnancy: Case Series from a Tertiary Care Centre in Western India

**DOI:** 10.7759/cureus.110727

**Published:** 2026-06-12

**Authors:** Sweta Shrivastava, Heena Rajput, Aayushi Thakkar

**Affiliations:** 1 Obstetrics and Gynaecology, Smt. Bhikiben Kanjibhai Shah Medical Institute and Research Centre (SBKS MIRC), Sumandeep Vidyapeeth Deemed to be University, Vadodara, IND

**Keywords:** acute kidney injury, extra-uterine pregnancy, laparoscopy, rudimentary horn pregnancy, ruptured tubal ectopic

## Abstract

Ectopic pregnancy is a potentially life-threatening gynecological emergency and an important cause of first-trimester maternal morbidity. It may present with a wide spectrum of clinical manifestations, ranging from asymptomatic early diagnosis to catastrophic rupture with hemodynamic instability. Prompt diagnosis and timely intervention are crucial for successful outcomes. We present a case series of six patients managed for ectopic pregnancy at a tertiary care centre with varied presentations and management approaches. The series included three cases of ruptured tubal ectopic pregnancy, one unruptured tubal ectopic pregnancy, one unruptured rudimentary horn ectopic pregnancy, and one case of impending tubal abortion. One patient with a ruptured tubal ectopic pregnancy presented in a hemodynamically unstable condition and underwent emergency laparotomy. The remaining five patients were managed laparoscopically. Diagnosis in all cases was established by clinical assessment, serum beta-human chorionic gonadotropin levels, and ultrasonography findings. Surgical management was individualized according to the patient’s clinical status, site of ectopic pregnancy, and intraoperative findings. Five patients had an uncomplicated postoperative recovery. One patient with a ruptured tubal ectopic pregnancy managed laparoscopically developed postoperative acute kidney injury, requiring further intensive management. Subsequent recovery was achieved with appropriate multidisciplinary care. All patients ultimately recovered satisfactorily. This case series highlights the varied clinical spectrum of ectopic pregnancy and emphasizes the importance of early diagnosis, individualized surgical management, and vigilant postoperative monitoring for optimal outcomes.

## Introduction

Ectopic pregnancy is defined as implantation of a fertilized ovum outside the endometrial cavity and remains one of the most common gynecological emergencies encountered in reproductive-age women [1]. It accounts for approximately 1%-2% of all pregnancies worldwide and is a significant cause of first-trimester maternal morbidity and mortality [2]. Delay in diagnosis or management may result in rupture and life-threatening intra-abdominal hemorrhage [3]. The most common site of implantation is the fallopian tube, although ectopic gestation may also occur in uncommon locations such as the rudimentary horn of a unicornuate uterus, ovary, cervix, cesarean scar or abdominal cavity [4].

The clinical presentation of ectopic pregnancy is highly variable, ranging from mild abdominal pain and vaginal spotting to acute abdomen, shock, and collapse in cases of rupture. Advances in transvaginal ultrasonography and serum β-human chorionic gonadotropin (β-hCG) estimation have improved the early diagnosis of ectopic pregnancy [5,6]. Management depends upon the patient’s hemodynamic status, site of implantation, extent of rupture, desire for future fertility, and available surgical expertise [7,8]. While laparoscopy is considered the preferred modality in hemodynamically stable patients due to faster recovery and reduced postoperative morbidity, laparotomy remains essential in unstable patients or when rapid control of hemorrhage is required [9].

This case series aims to present the varied clinical presentations, surgical management strategies, and outcomes of six patients diagnosed with ectopic pregnancy at a tertiary care center.

## Materials and methods

This retrospective case series was conducted in the Department of Obstetrics and Gynecology at Dhiraj Hospital, SBKS Medical Institute and Research Centre, Vadodara, Gujarat, over a period of three months, from January 2026 to March 2026. The study included six consecutive patients who were diagnosed and managed as ectopic pregnancy during the study period.

Inclusion Criteria

The inclusion criteria were as follows: (1) Women diagnosed with ectopic pregnancy based on clinical, biochemical, and ultrasonographic findings; (2) patients managed either surgically or medically at the center during the study period; and (3) cases with complete clinical records, operative findings, and follow-up details available for review.

Exclusion Criteria

The exclusion criteria were as follows: (1) Patients diagnosed with pregnancy of unknown location without confirmation of ectopic pregnancy; (2) patients referred after definitive management elsewhere; and (3) heterotopic pregnancies and non-gynecological causes of acute abdomen.

Diagnosis of ectopic pregnancy was established based on a combination of clinical suspicion, positive pregnancy test, serum β-hCG (human chorionic gonadotropin) estimation, and ultrasonographic findings. Surgical management was individualized according to patient stability, site of ectopic gestation, and intraoperative findings. Patient confidentiality and anonymity were maintained throughout the study.

Outcome Measures

The primary outcome of the study was successful management of ectopic pregnancy without major maternal morbidity or mortality.

The secondary outcomes included: (1) Clinical presentation patterns of ectopic pregnancy; (2) type of ectopic pregnancy encountered; (3) surgical procedures performed; (4) requirement of blood transfusion; (5) duration of hospital stay, and (6) immediate postoperative recovery and complications.

## Results

Case 1

**Figure 1:**
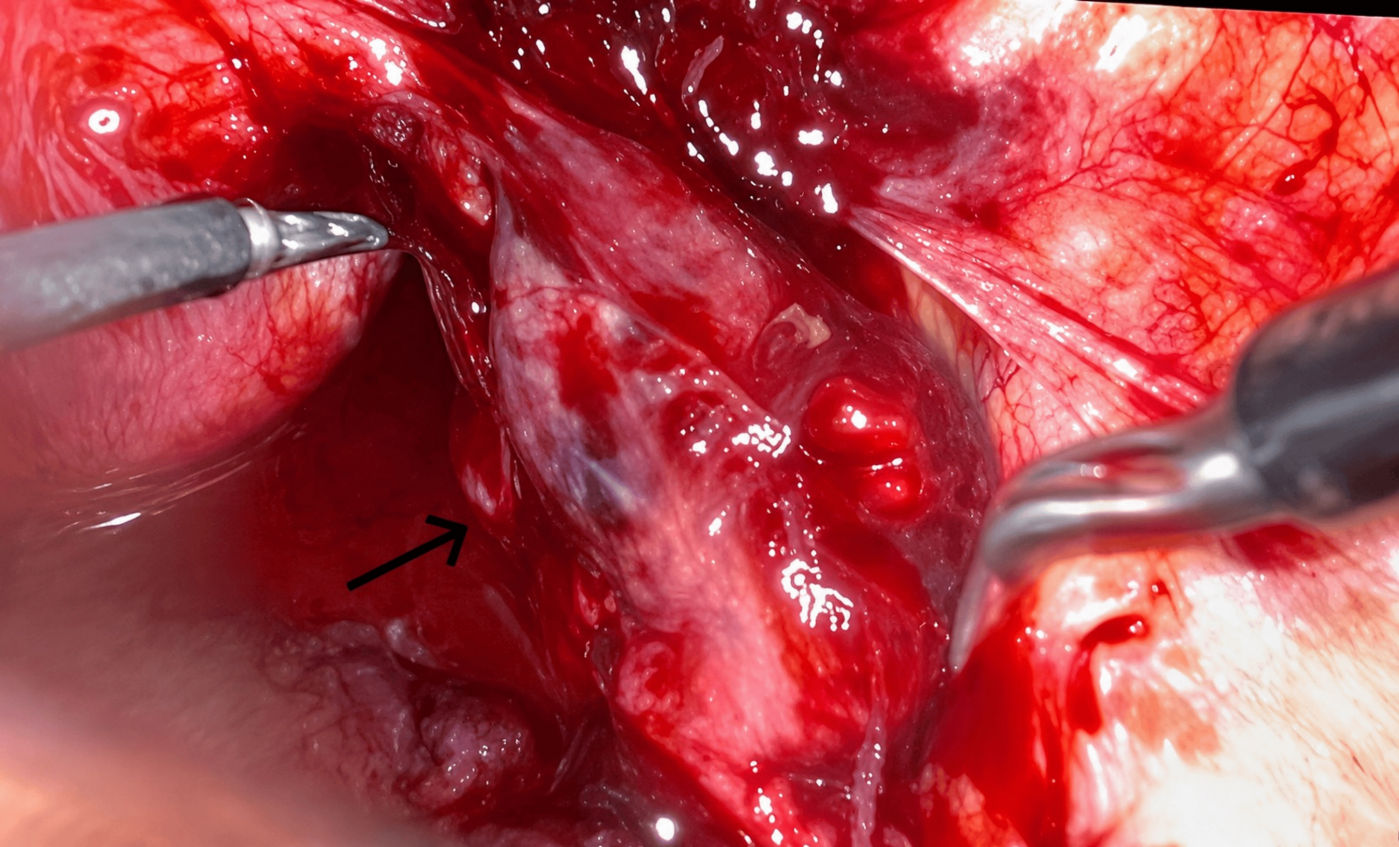
Case 1 - Right ruptured ectopic pregnancy. Laparoscopic image showing distended and congested right fallopian tube (black arrow) with hemoperitoneum suggestive of ruptured ectopic pregnancy.

A 23-year-old gravida 3 para 1 abortion 1 presented with two months of amenorrhoea and lower abdominal pain. Gestational age was eight weeks and four days. On admission, she was tachycardic (pulse 120/min) with maintained blood pressure. Serum β-hCG was 6581 mIU/mL. Ultrasonography revealed a 5.5×10 cm solid cystic lesion in the right adnexa with mild to moderate hemoperitoneum, suggestive of right ruptured tubal ectopic pregnancy. She underwent emergency laparoscopic right salpingectomy followed by endometrial curettage. Estimated intraoperative blood loss was 900 ml. Hemoglobin on admission was 7.6 g/dL. She received one unit of packed red blood cell transfusion followed by 1 g ferric carboxy maltose infusion. Postoperative recovery was uneventful, and she was discharged on postoperative day 3 (Figure 1). 

Case 2

**Figure 2:**
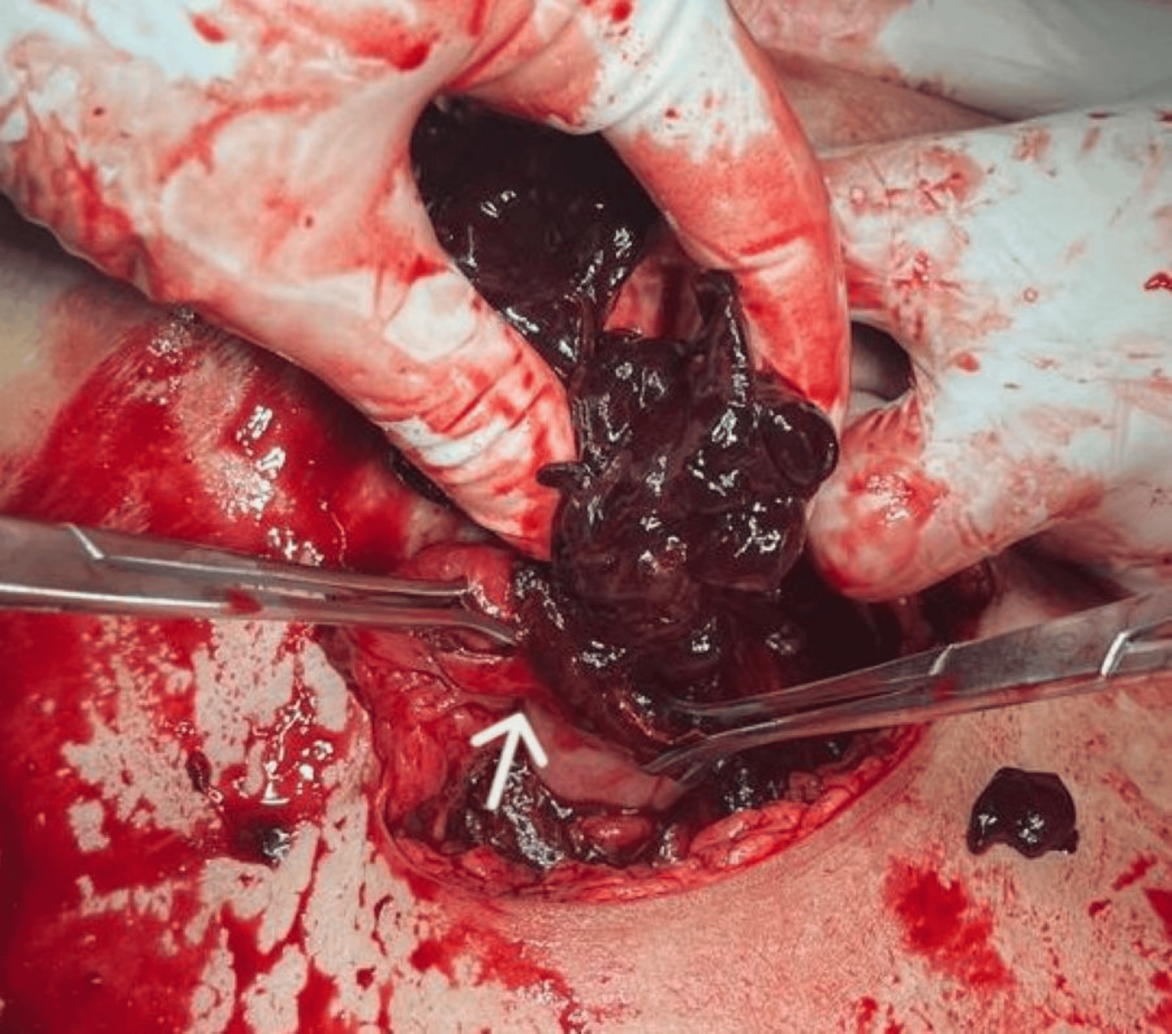
Case 2 - Laprotomy for ruptured tubal ectopic pregnancy Intraoperative view during emergency laparotomy demonstrating ruptured right fallopian tube (white arrow) with hemoperitoneum and removal of products of conception.

A 23-year-old gravida 2 para 1 with a previous lower segment caesarean section was referred from a community health center with a hemoglobin of 4.6 g/dL. She presented with vomiting for two days and lower abdominal pain for one day, with six weeks and six days of amenorrhoea. On admission, she was febrile (temperature 102°F) and tachycardic (pulse 128/min) with blood pressure 120/90 mmHg. Based on clinical and intraoperative findings, she was diagnosed with a right ruptured tubal ectopic pregnancy with very severe anemia. She underwent emergency exploratory laparotomy with right salpingectomy and endometrial curettage. Intraoperatively, a ruptured right tubal ectopic pregnancy was confirmed (Figure 2).

The estimated blood loss was 600 mL. She received two units of packed red blood cells and four units of fresh frozen plasma perioperatively. Her postoperative course was uneventful, and got discharged on postoperative day three.

Case 3

**Figure 3:**
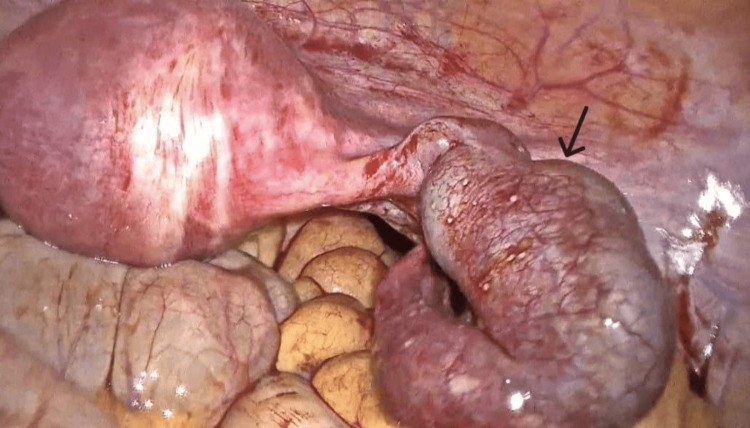
Case 3 - Unruptured tubal ectopic pregnancy Laparoscopic image showing distended and congested right fallopian tube (black arrow) suggestive of unruptured ectopic pregnancy.

A 26-year-old primigravida presented with one and a half months of amenorrhoea and a positive urine pregnancy test. She was asymptomatic at presentation with no specific complaints. Her vital parameters were stable. Ultrasonography revealed a thick-walled complex cystic structure with a mean sac diameter of 0.97 cm, without a fetal pole or yolk sac, suspicious of a right-sided ovarian ectopic pregnancy. Serum β-hCG was 12,229 mIU/mL. Based on further evaluation and intraoperative findings, a diagnosis of right unruptured adnexal tubal ectopic pregnancy was made (Figure 3).

She underwent laparoscopic right salpingectomy followed by endometrial curettage. Intraoperatively, the right fallopian tube was distended with ectopic gestation involving the ampullary region, while the right ovary appeared completely normal. The estimated blood loss was 50 mL. Her postoperative recovery was uneventful, and she was discharged on postoperative day three.

Case 4

**Figure 4:**
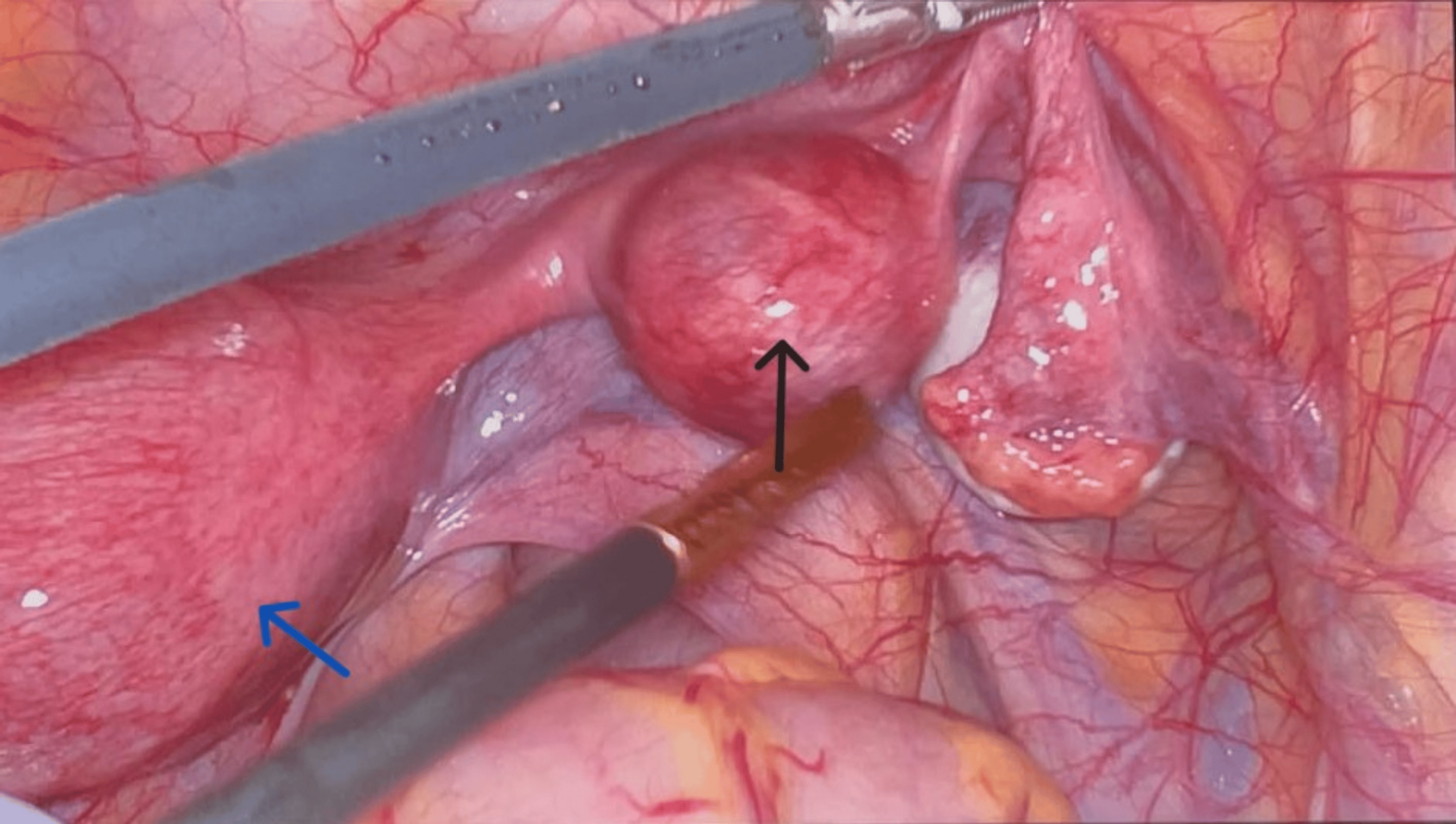
[Case 4] Rudimentary horn ectopic pregnancy Laparoscopic view demonstrating a rudimentary uterine horn (black arrow) attached to a unicornuate uterus (blue arrow).

A 22-year-old gravida 2 abortion 1 presented with two months of amenorrhoea and a positive urine pregnancy test with stable vitals. Ultrasonography showed a uterus with an endometrial thickness of 14 mm and no evidence of an intrauterine gestational sac. A gestational sac-like anechoic structure with a mean sac diameter of 1.2 cm was noted in the right adnexa. A yolk sac and fetal pole were visualized, with a crown-rump length of 0.76 cm, corresponding to six weeks and five days of gestation, with cardiac activity present. She was provisionally diagnosed with an unruptured right adnexal ectopic pregnancy and planned for laparoscopy. Intraoperatively, a 3×2 cm mass with a thick muscular wall was identified on the right lateral side of the uterus and medial to the right fallopian tube and ovary; the right tube and ovary were found to be normal, consistent with a right rudimentary horn pregnancy (Figure 4).

**Figure 5:**
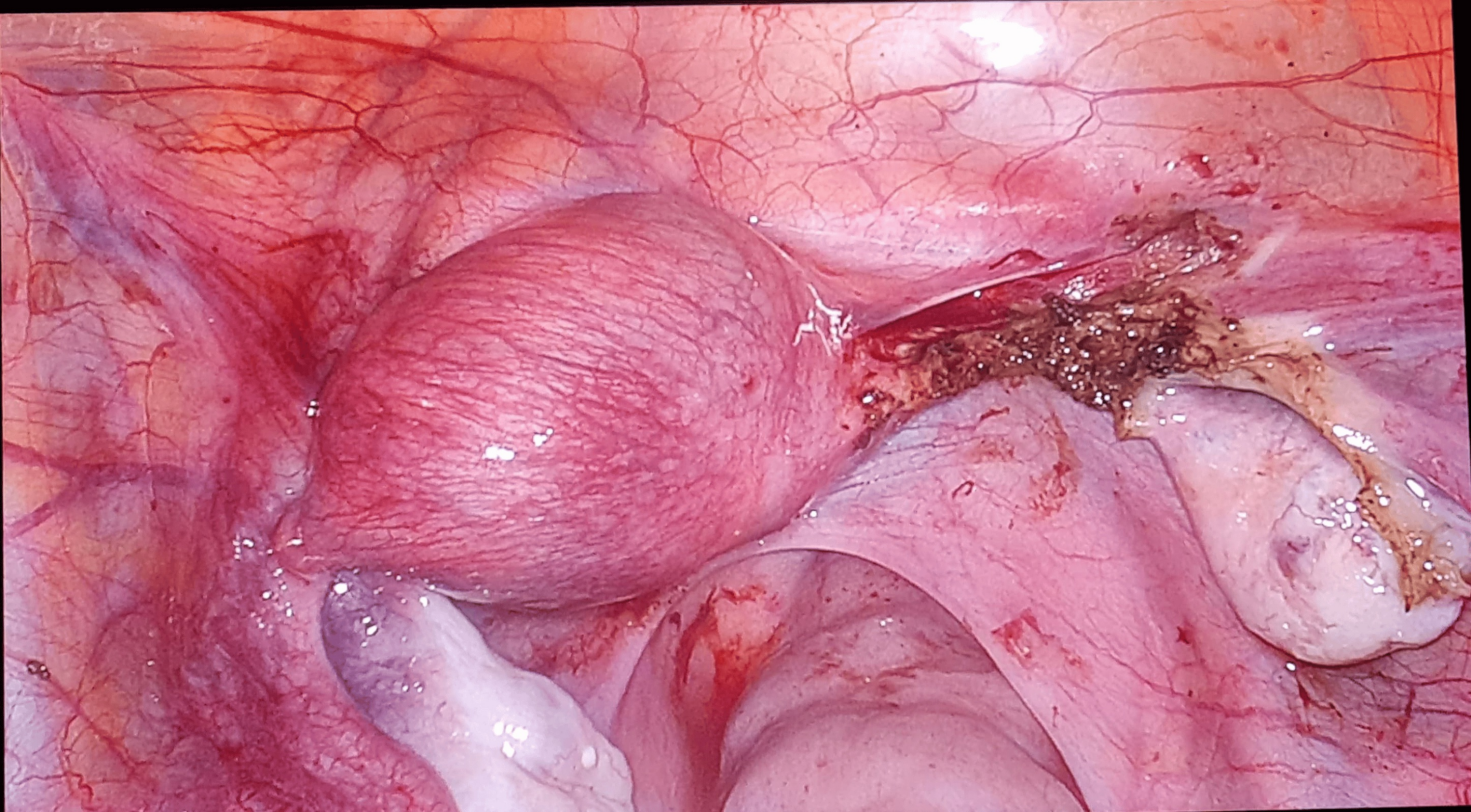
Case 4 - Post-rudimentary horn ectopic excision Post-Rudimentary horn excision laparoscopic image showing the remaining Unicornuate uterus.

She underwent laparoscopic excision of the right rudimentary horn ectopic pregnancy with right salpingectomy followed by endometrial curettage (Figure 5).

**Figure 6:**
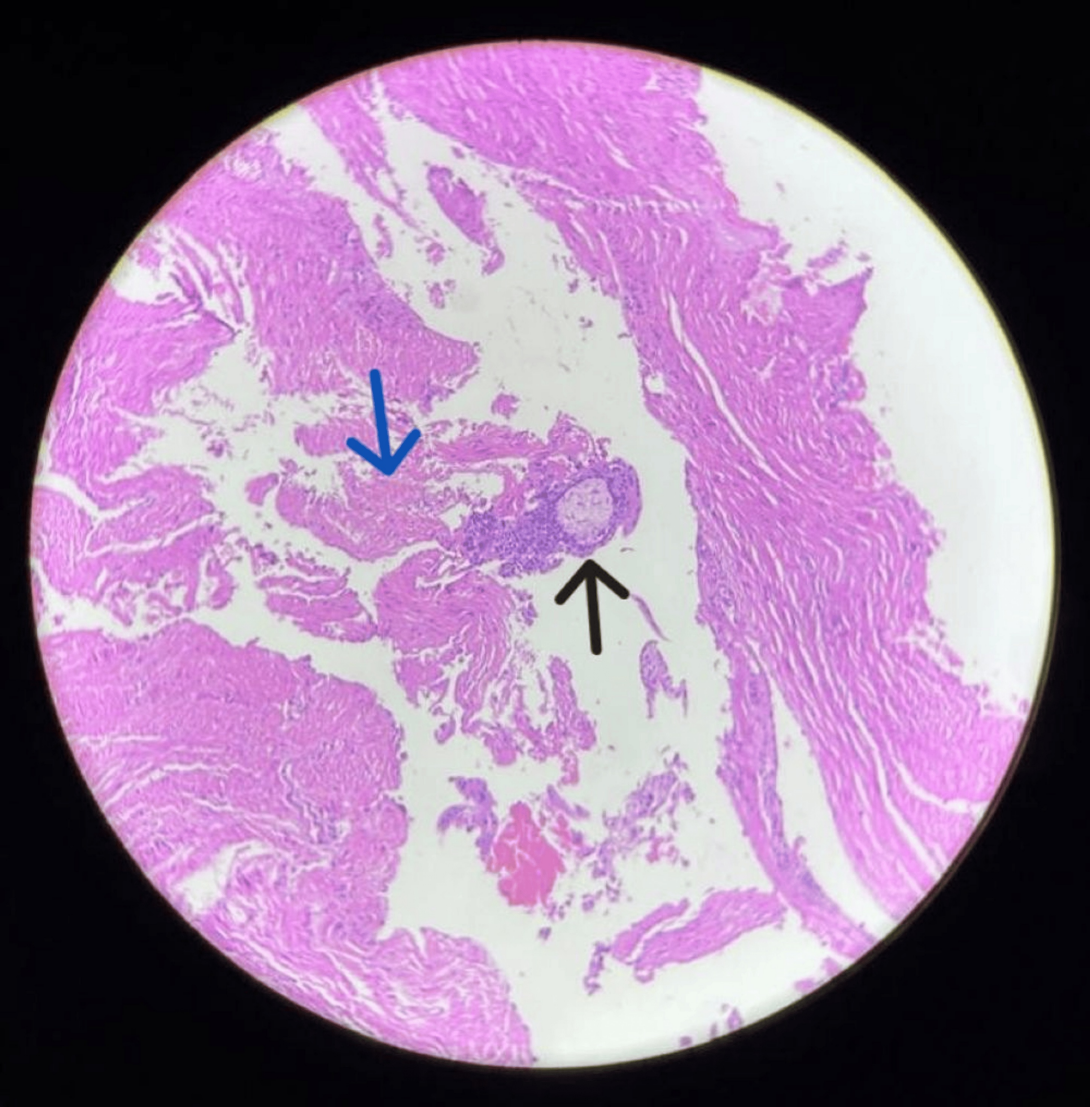
Case 4 - Histopathology image Histopathological section (hematoxylin and eosin stain) showing chorionic villi (black arrow) embedded within surrounding myometrial tissue (blue arrow), confirming rudimentary horn pregnancy.

The estimated blood loss was 20 mL. Her postoperative recovery was uneventful, and she was discharged on postoperative day 3. Histopathological examination confirmed the presence of chorionic villi within myometrial tissue, establishing the diagnosis of rudimentary horn ectopic pregnancy (Figure 6).

Case 5

**Figure 7:**
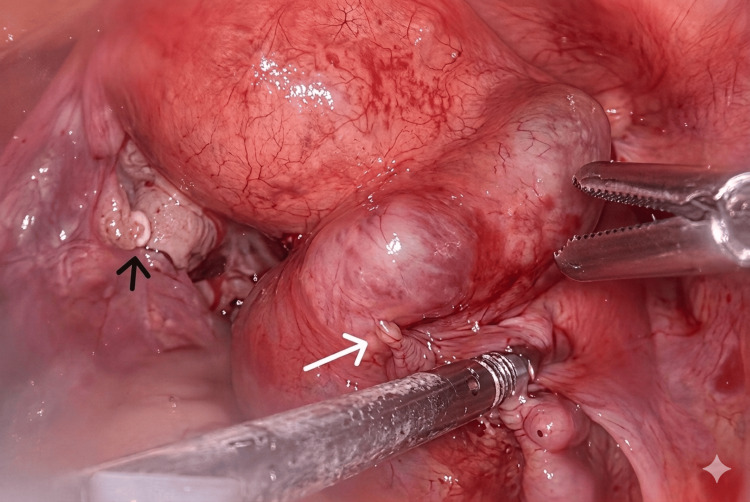
Case 5 - Post-sterilization tubal ectopic pregnancy Laparoscopic image showing distended and congested right fallopian tube (white arrow) suggestive of ectopic pregnancy. Fallope’s ring is visible (black arrow), indicating previous tubal ligation.

A 30-year-old gravida 5 para 3 abortion 1 with all previous normal vaginal deliveries and a history of laparoscopic tubal ligation performed three years earlier, presented with right-sided lower abdominal pain for eight days. She had been admitted to another hospital for three days, where she was diagnosed with an unruptured right tubal ectopic pregnancy and received single-dose methotrexate as medical management. However, due to persistent pain and unwillingness for further medical management, she presented to our center. She was clinically stable on admission. Ultrasonography revealed a uterus with an endometrial thickness of 5.1 mm and no intrauterine gestational sac. A heterogeneously hyperechoic lesion measuring 2.7×3.1×2.5 cm was noted in the right adnexa, possibly within the right fallopian tube, with no internal vascularity on Doppler study. Serum β-hCG was 645 mIU/mL on day 1 and 680 mIU/mL after 48 hours. A diagnosis of right unruptured tubal ectopic pregnancy was made. She underwent laparoscopic bilateral salpingectomy followed by endometrial curettage. Intraoperatively, the right fallopian tube was distended, measuring approximately 2 × 5 cm, with minimal hemoperitoneum present (Figure 7).

As the patient had completed family size and previously undergone sterilization, bilateral salpingectomy was performed. Estimated blood loss was 50-100 mL. Her postoperative recovery was uneventful, and she was discharged on postoperative day 3.

Case 6

**Figure 8:**
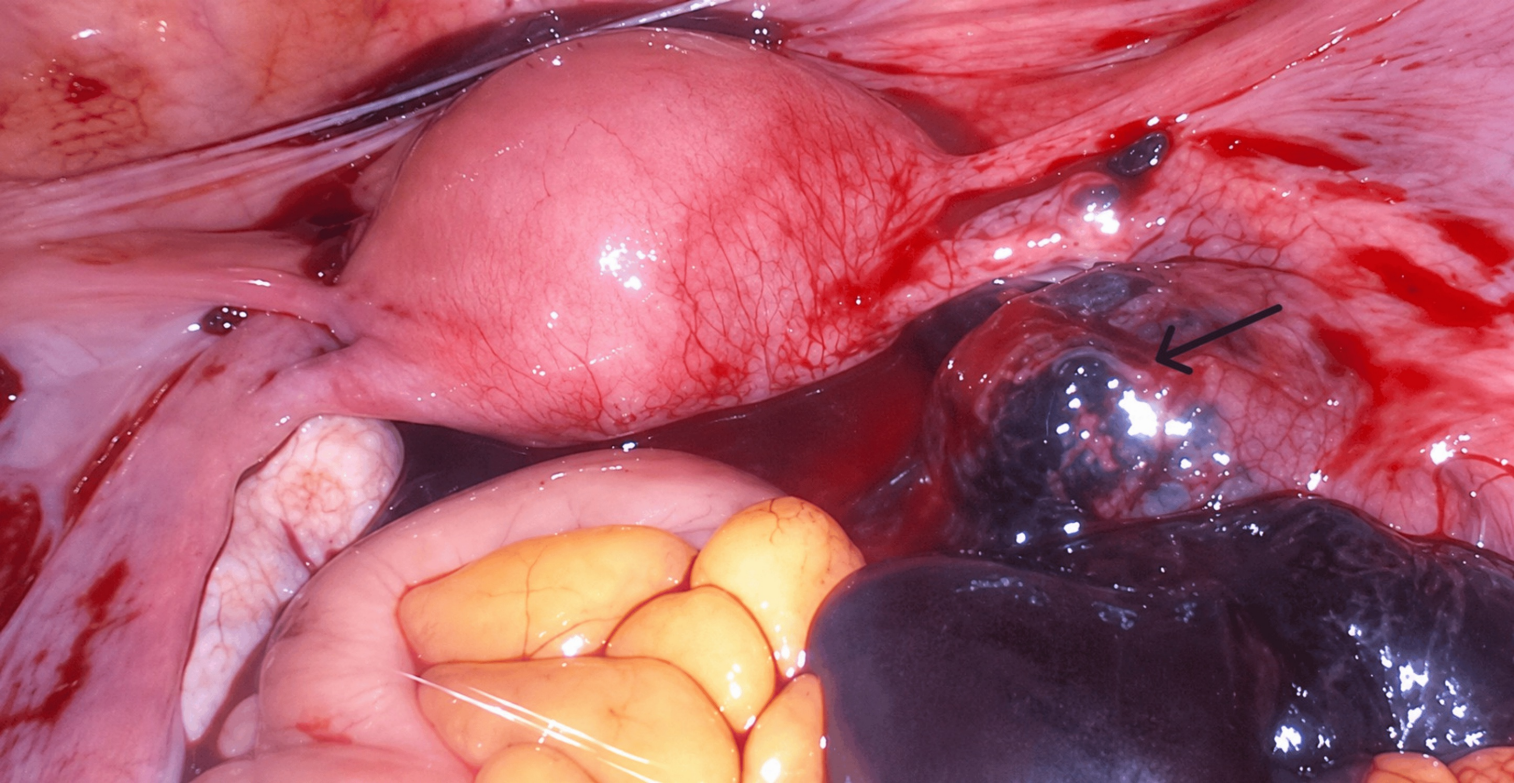
Case 6 - Ruptured tubal ectopic pregnancy Laparoscopic image showing uterus and ruptured right fallopian tube (black arrow) with associated hemoperitoneum and blood clots.

**Figure 9:**
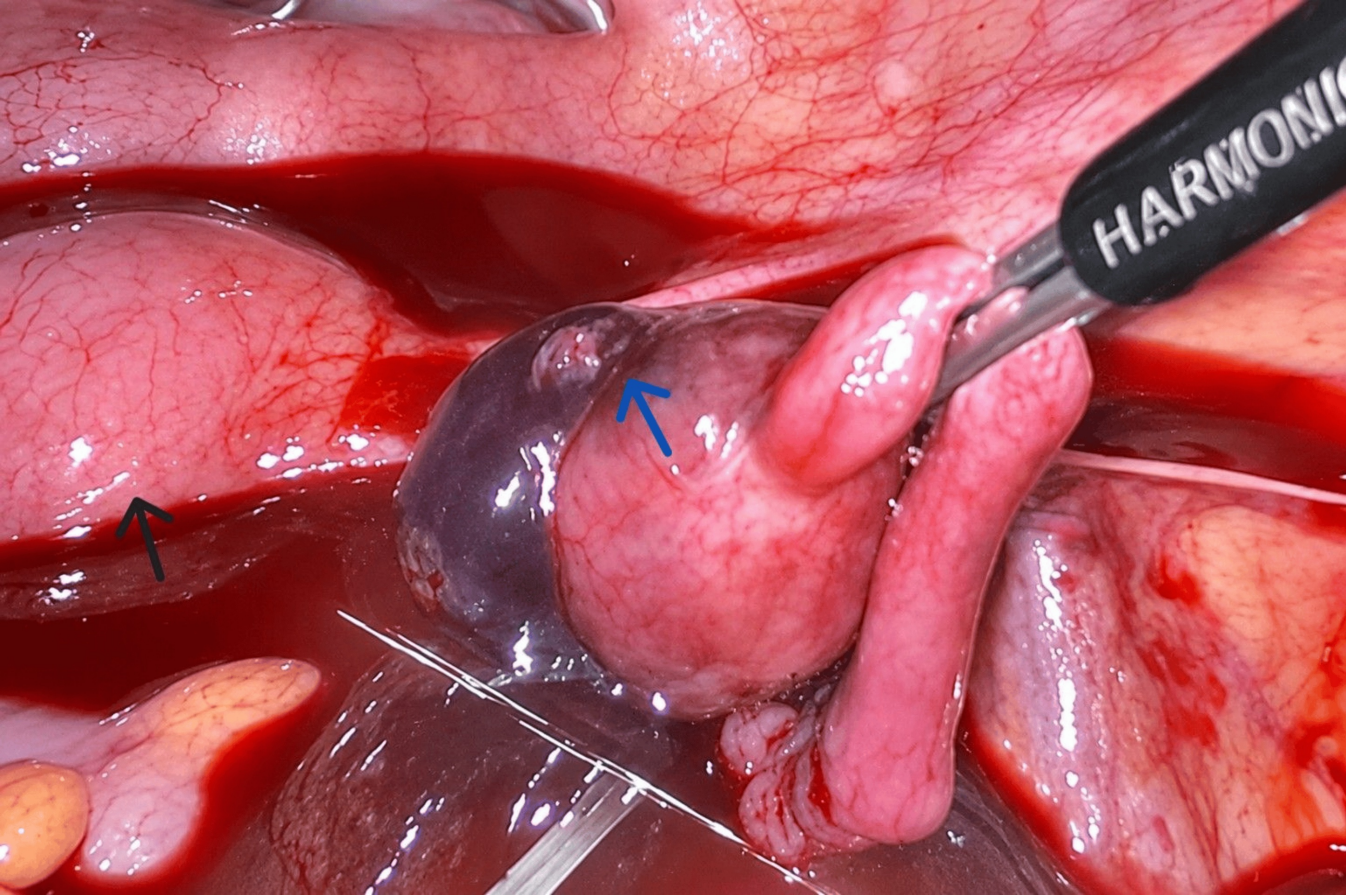
Case 6 - Post-sterilization ruptured tubal ectopic pregnancy Laparoscopic image showing uterus (black arrow) and ruptured right fallopian tube (blue arrow) with associated hemoperitoneum, consistent with ruptured ectopic pregnancy.

A 32-year-old gravida 3 para 2 with a history of interval mini laparotomy tubal ligation performed two years prior, presented with two months of amenorrhoea and acute onset abdominal pain for one day. The urine pregnancy test was positive. On examination, she had marked pallor, with stable vital parameters. Ultrasonography revealed a right adnexal gestational sac with a crown-rump length of 1.2 cm (seven weeks three days) with cardiac activity, along with mild hemoperitoneum, suggestive of right ruptured tubal ectopic pregnancy. Serum β-hCG was 83,154 mIU/mL. She underwent emergency laparoscopic bilateral salpingectomy. Intraoperatively, a ruptured right tubal ectopic pregnancy with hemoperitoneum and clots was noted (Figures 8, 9).

Given the history of prior sterilization, bilateral salpingectomy was performed. The estimated blood loss was 500-600 mL. Hemoglobin on admission was 8.9 g/dL, and she received one unit of packed red blood cells intraoperatively. In the postoperative period, the patient developed acute kidney injury (AKI) characterized by oliguria and rising renal parameters from postoperative day 1 (creatinine 2.8 mg/dL, urea 70 mg/dL). Urine output declined to 450-700 mL/day over the first three postoperative days. She also developed fever spikes on postoperative days 2 and 3, necessitating escalation of care.

The patient was shifted to the intensive care unit on postoperative day 2 and managed with a multidisciplinary approach involving a nephrologist and an intensivist. Management included fluid restriction (1-1.5 L/day), intravenous meropenem, diuretics (torsemide), supportive care, and blood transfusions (total four units of packed red blood cells). Hemodialysis was considered but ultimately not required. From postoperative day five, urine output improved significantly (>2000 mL/day), although renal function continued to worsen transiently, with peak serum creatinine reaching 7.7 mg/dL on postoperative day seven. Gradual improvement was noted thereafter, with creatinine declining to 3.0 mg/dL by postoperative day 11. The patient remained hemodynamically stable and afebrile. She was discharged on postoperative day 12 in stable condition with improving renal parameters.

Table 1 shows the summary of clinical presentation, management, and outcomes in six cases of ectopic pregnancy. 

**Table 1:** Clinical profile, management, and outcomes of ectopic pregnancy cases. Abbreviations: GA: Gestational age; Hb: hemoglobin; MTX: methotrexate; PRBC: packed red blood cells; AKI: acute kidney injury.

Case	Age (Years)	Obstetric History	GA (weeks)	Hb (g/dL)	β-hCG (mIU/mL)	Presentation	Diagnosis	Management	Blood Loss / Transfusion	Complications
1	23	G3P1L1A1	8.4	7.6	6581	Pain in abdomen, amenorrhoea	Ruptured right tubal ectopic	Laparoscopic right salpingectomy + endometrial curettage	~900 mL, 1 PRBC+ Parenteral Iron	Nil
2	23	G2P1L1	6.6	4.6	NA	Amenorrhoea, Pain in abdomen, vomiting, fever	Ruptured right tubal ectopic with severe anemia	Laparotomy + right salpingectomy + endometrial curettage	~600 mL, 2 PRBC + 4 Fresh Frozen Plasma (FFP)	Nil
3	26	Primi	6	12.1	12,229	Amenorrhoea	Unruptured right tubal ectopic	Laparoscopic right salpingectomy + endometrial curettage	~50 mL	Nil
4	22	G2A1	7.6	11.5	NA	Amenorrhoea	Unruptured Rudimentary horn ectopic	Laparoscopic rudimentary horn excision + right salpingectomy + endometrial curettage	~20 mL	Nil
5	30	G5P3L3A1 (post TL)	5.3	11.9	645→680	Pain in abdomen, failed MTX	Unruptured right tubal ectopic	Laparoscopic bilateral salpingectomy + endometrial curettage	50-100 mL	Nil
6	32	G3P2L2 (post TL)	8.2	8.9	83,154	Pain in abdomen, amenorrhoea	Ruptured right tubal ectopic	Laparoscopic bilateral salpingectomy + endometrial curettage	500-600 mL, 4 PRBC	AKI

## Discussion

Ectopic pregnancy remains a significant cause of first-trimester maternal morbidity and continues to pose diagnostic and therapeutic challenges. In the present case series, tubal ectopic pregnancy constituted the majority of cases, with one case of rudimentary horn pregnancy highlighting an uncommon but clinically important variant [4].

The clinical presentation of ectopic pregnancy is often variable and non-specific. In our series, most patients presented with amenorrhoea and abdominal pain, which is consistent with findings reported in the recent literature [5]. One patient was asymptomatic and diagnosed incidentally on ultrasonography, emphasizing the importance of early imaging in suspected cases. Another patient presented with fever and very severe anemia, indicating delayed presentation and advanced disease.

In our series, two patients had a history of tubal ligation, representing failure of sterilization. Post-sterilization ectopic pregnancies are well recognized and may occur due to tubal recanalization or fistula formation, necessitating a high index of suspicion even in women with prior sterilization [9]. Early diagnosis relies on a combination of serum β-hCG estimation and transvaginal ultrasonography, which remain the cornerstone of evaluation [10,11]. In our study, ultrasonography was crucial in identifying adnexal masses and hemoperitoneum. However, one case initially suspected to be an ovarian ectopic was confirmed intraoperatively as a tubal ectopic pregnancy, highlighting the diagnostic challenges associated with atypical imaging findings.

Management of ectopic pregnancy depends on the patient’s hemodynamic status, site of implantation, and available expertise [12,13]. In the present series, laparoscopy was successfully performed in five out of six patients, including selected cases of ruptured ectopic pregnancy, demonstrating its safety and feasibility in hemodynamically stable patients. However, laparotomy remains essential in cases of hemodynamic instability or when rapid control of hemorrhage is required [14], as observed in one of our patients. Individualized management is essential in ectopic pregnancy. One patient in our series had received methotrexate therapy at another center but required surgical intervention due to persistent symptoms, highlighting the importance of careful patient selection and follow-up during medical management [15]. Additionally, in patients with completed family size and prior sterilization, bilateral salpingectomy was performed as definitive management. Rudimentary horn pregnancy is a rare form of ectopic gestation associated with a high risk of rupture due to the limited distensibility of the horn [16]. In our series, the rudimentary horn ectopic was successfully managed laparoscopically, emphasizing the role of minimally invasive surgery even in rare presentations.

Postoperative outcomes were favorable in the majority of cases. However, one patient developed acute kidney injury (AKI) in the postoperative period, which is an uncommon but serious complication. The patient presented with oliguria and progressively rising renal parameters, requiring intensive care and multidisciplinary management. AKI in such cases may be multifactorial, including hypovolemia, sepsis, or perioperative hemodynamic changes [17]. Early recognition and prompt supportive management resulted in recovery without the need for dialysis. This highlights the importance of vigilant postoperative monitoring and timely intervention to prevent adverse outcomes.

Overall, this case series highlights the diverse clinical spectrum, diagnostic challenges, and evolving management strategies in ectopic pregnancy, emphasizing the role of early diagnosis, minimally invasive surgery, and individualized patient care.

Limitations of the study

This case series is limited by the small sample size and single-centre design. Long-term follow-up and fertility outcomes were not assessed. Additionally, the varied clinical presentations and management approaches limited uniform comparison between cases.

Learning points

Ectopic pregnancy can present with a wide spectrum of clinical presentations, ranging from asymptomatic cases detected incidentally to life-threatening emergencies with severe anemia and hemodynamic compromise. A high index of suspicion should be maintained in women with amenorrhea and abdominal pain, even in those with a history of tubal sterilization, as post-sterilization ectopic pregnancy remains a recognized clinical entity.

Serum β-hCG estimation combined with transvaginal ultrasonography remains the cornerstone of early diagnosis; however, atypical imaging findings may necessitate intraoperative confirmation. Laparoscopic management is safe and effective in appropriately selected hemodynamically stable patients, including certain cases of ruptured ectopic pregnancy, while laparotomy remains essential in unstable patients.

Rare ectopic pregnancies, such as rudimentary horn pregnancy, require early recognition and prompt intervention because of their high risk of rupture and associated morbidity. Careful postoperative monitoring is essential for the early detection and management of complications, including uncommon but potentially serious conditions such as acute kidney injury.

## Conclusions

Ectopic pregnancy continues to pose diagnostic and therapeutic challenges due to its varied clinical presentation. This case series illustrates the use of different diagnostic modalities and individualized management strategies, including both laparoscopy and laparotomy, based on clinical circumstances. Timely intervention and careful postoperative monitoring remain essential for optimizing outcomes and detecting complications.
